# The ubiquitin ligase Ozz decreases the replacement rate of embryonic myosin in myofibrils

**DOI:** 10.14814/phy2.15003

**Published:** 2021-08-26

**Authors:** Emi Ichimura, Koichi Ojima, Susumu Muroya, Takahiro Suzuki, Ken Kobayashi, Takanori Nishimura

**Affiliations:** ^1^ Research Faculty of Agriculture Graduate School of Agriculture Hokkaido University Sapporo Japan; ^2^ Muscle Biology Research Unit Division of Animal Products Research Institute of Livestock and Grassland Science NARO Tsukuba Japan; ^3^ Department of Bioresource Sciences Faculty of Agriculture Kyushu University Fukuoka Japan

**Keywords:** myofibril, myosin, skeletal muscle, thick filament, ubiquitin ligase, ubiquitin–proteasome system

## Abstract

Myosin, the most abundant myofibrillar protein in skeletal muscle, functions as a motor protein in muscle contraction. Myosin polymerizes into the thick filaments in the sarcomere where approximately 50% of embryonic myosin (Myh3) are replaced within 3 h (Ojima K, Ichimura E, Yasukawa Y, Wakamatsu J, Nishimura T, Am J Physiol Cell Physiol 309: C669‐C679, 2015). The sarcomere structure including the thick filament is maintained by a balance between protein biosynthesis and degradation. However, the involvement of a protein degradation system in the myosin replacement process remains unclear. Here, we show that the muscle‐specific ubiquitin ligase Ozz regulates replacement rate of Myh3. To examine the direct effect of Ozz on myosin replacement, eGFP‐Myh3 replacement rate was measured in myotubes overexpressing Ozz by fluorescence recovery after photobleaching. Ozz overexpression significantly decreased the replacement rate of eGFP‐Myh3 in the myofibrils, whereas it had no effect on other myosin isoforms. It is likely that ectopic Ozz promoted myosin degradation through increment of ubiquitinated myosin, and decreased myosin supply for replacement, thereby reducing myosin replacement rate. Intriguingly, treatment with a proteasome inhibitor MG132 also decreased myosin replacement rate, although MG132 enhanced the accumulation of ubiquitinated myosin in the cytosol where replaceable myosin is pooled, suggesting that ubiquitinated myosin is not replaced by myosin in the myofibril. Collectively, our findings showed that Myh3 replacement rate was reduced in the presence of overexpressed Ozz probably through enhanced ubiquitination and degradation of Myh3 by Ozz.

## Graphical abstract

In our model, ubiquitination of embryonic myosin (Myh3) is enhanced in the cytosol of Ozz‐overexpressed myotubes. Ubiquitinated Myh3 is not incorporated into the thick filaments but degraded through the proteasome system, leading to slow Myh3 replacement rate in the thick filaments.

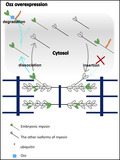

## 1 INTRODUCTION

Because of its remarkable plasticity, skeletal muscle tissue can modify its volume to adapt to exercise, disease, development, and aging (Lecker et al., 2006; Nury et al., 2007). Skeletal muscle tissue is composed of bundles of myofibers, which contain highly organized structural components named myofibrils. A single myofiber contains approximately 2,000 of myofibrils in untrained adult humans (Placzek & Boyce, 2016). Myofibrillar proteins, of which there are more than 20 types, are organized into repeated minimal contraction units referred to as sarcomeres (Clark et al., 2002; Henderson et al., 2018). Muscle tissue size is determined by the volume of each muscle fiber, which reflects the number of myofibrils. Therefore, muscle hypertrophy and atrophy depend on increase and decrease of the number of myofibrils, respectively, and, muscle volume is modulated by the balance between the synthesis and degradation of myofibrillar proteins.

Myosin, an abundant myofibrillar protein in the skeletal muscle, functions as a motor protein in muscle contraction. Myosin is composed of two heavy chains and four light chains, and polymerizes into filaments under physiological ionic strength conditions (Craig & Woodhead, 2006; Davis, 1988). In myofibrils, approximately 300 myosin molecules form a single thick filament with myosin‐associated proteins such as connectin/titin, myomesin, and myosin‐binding protein C (MybpC) (Kontrogianni‐Konstantopoulos et al., 2009; Labeit & Titins, 1995; Obermann et al., 1997). Each sarcomere contains thick and thin filaments that are arranged in a regular pattern; thin filaments are composed of actin, troponin complex, tropomyosin, and nebulin (Prill & Dawson, 2020). The interaction of myosin in the thick filaments with actin in the thin filaments induces muscle contraction, coupled with ATP hydrolysis by myosin (Walklate et al., 2016).

Skeletal muscle is classified into two categories according to the velocity of muscle contraction, i.e., slow and fast types. This feature is attributed to the expression of myosin heavy chain (Myh) isoforms with different rates of ATPase activity and muscle contraction velocity in each myofiber (Resnicow et al., 2010; Walklate et al., 2016). Slow type muscle fibers express Myh7, whereas fast type myofibers express Myh1, Myh2, and/or Myh4. Although slow and fast types of Myhs are dominantly expressed in adult skeletal muscles, other Myh isoforms such as embryonic (Myh3) and neonatal (Myh8) types are expressed during muscle development. Fast type of Myhs gradually becomes predominant a few days after birth in mice, which causes the down‐regulation of Myh3 and Myh8, resulting in Myh isoform shift in myofibrils (Schiaffino et al., 2015).

Proteins are regularly degraded to maintain protein quality and quantity *in vivo*. One of the main degradation systems in skeletal muscle is the ubiquitin–proteasome system (UPS), which selectively degrades target proteins (Lecker et al., 2006; Passmore & Barford, 2004). In the UPS, ubiquitin (Ub) is activated by the E1 ubiquitin‐activating enzymes in an ATP‐dependent manner and is transferred to an E2 ubiquitin‐conjugating enzyme. The E3 ubiquitin ligase which selectively recognizes substrate proteins recruits the E2, catalyzing the transfer of Ub to a lysine (K) residue of the substrate (Passmore & Barford, 2004). Polyubiquitination on the substrate protein is classified for degradation by the proteasome (Huang & Zhang, 2020).

In striated muscle, muscle ring finger protein 1 (Murf1) and muscle atrophy F‐box (MAFbx) are muscle‐specific E3s that are up‐regulated under conditions of muscle atrophy (Bodine et al., 2001). Murf1 is a member of the Murf family, which ubiquitinates multiple myofibrillar proteins containing the slow and fast types of Myh (Cohen et al., 2009; Fielitz et al., 2007). Potential target proteins of MAFbx in sarcomeres include Myh, vimentin, and desmin (Lokireddy et al., 2011, 2012), in addition to its well‐known substrates MyoD and eukaryotic translation initiation factor 3 (Lagirand‐cantaloube et al., 2008; Tintignac et al., 2005). Neuralized E3 ubiquitin protein ligase 2 (Neurl2) which is referred to as Ozz, another muscle‐specific E3, is up‐regulated during myotube formation (Campos et al., 2010). Ozz, a member of the suppressor of cytokine signaling family, functions as an E3 in a complex containing Elongin B/C, Rbx1, and Cullin 5 (Kile et al., 2002). Ozz ubiquitinates Myh3 (Campos et al., 2010), and knockdown of Ozz causes defects in myofibril formation (Nastasi et al., 2004). Therefore, Ozz is a critical skeletal muscle E3 at the embryonic stage.

In a previous study, we investigated the myosin replacement rate in myofibrils using fluorescence recovery after photobleaching (FRAP) technique in myotubes expressing enhanced green fluorescence protein (eGFP) tagged Myh3. The half‐life of myosin is approximately 5 days as measured by the radioisotope technique in muscle cells (Rubinstein et al., 1976; Zak et al., 1977); however, we found that myosin molecules in myofibrils are replaced more frequently, i.e., approximately 50% of myosin molecules in myofibrils are replaced within 3 h (Ojima et al., 2015). The myosin in myofibrils is replaced by another myosin molecule that is newly synthesized and/or derived from a cytosolic pool of myosin (Ojima et al., 2015, 2017). However, the role of protein degradation system in myosin replacement has not been investigated. In this study, we examined the effect of Ozz overexpression on eGFP‐Myh3 replacement rate. The result showed that Ozz up‐regulation promoted the ubiquitination and degradation of myosin, thereby decreasing the rate of embryonic myosin replacement. The present findings underscore the importance of the UPS in myosin replacement in skeletal muscle cells.

## 2 MATERIALS AND METHODS

### 2.1 Experimental animals

All experiments were performed using primary muscle cells from chick embryos. Experimental animals were cared for as outlined in the guidelines of Hokkaido University and NARO for the care and use of laboratory animals. This study was approved by the two committees.

### 2.2 Cell culture and transfection

Skeletal muscle cells were isolated from the pectoral muscles of 11‐day‐old chick embryos without sex determination as described previously (Ojima et al., 2015). After removing connective tissue under a stereomicroscope, minced muscles were incubated with a 0.025% trypsin solution for 25 min at 37℃. Trypsinization of the muscle slurry was stopped by addition of growth medium [10% (v/v) horse serum (Thermo Fisher Scientific), 10% (v/v) embryo extract, and 1% (v/v) penicillin–streptomycin–glutamine ×100 (Thermo Fisher Scientific) in minimum essential medium (Thermo Fisher Scientific)]. The cell suspension was centrifuged at 600 *g* for 5 min, and the precipitate was dissolved in 5‐ml growth medium and filtered through 100‐ and 40‐µm cell strainers (CORNING). Cells were cultured on an uncoated 60‐mm dish at 37℃ for 50 min to remove contaminating non‐muscle cells. The non‐adherent cell fraction containing skeletal muscle cells was seeded at a density of 7500–10,000 cells/cm^2^ on dishes coated with poly‐L‐Lysine (Sigma‐Aldrich) and collagen Type Ⅰ‐A (Nitta Gelatin). One day after seeding, the cells were transfected with expression vectors using Lipofectamine®︎ LTX and Plus reagents (Thermo Fisher Scientific). The growth medium was replaced by differentiation medium [11% (v/v) horse serum, 3% (v/v) embryo extract, and 1% (v/v) penicillin–streptomycin–glutamine ×100 in minimum essential medium] 1 day after transfection or after reaching 70% cell confluence. The differentiation medium was replaced every 2 days. Once myotubes were formed, 10‐µM cytosine arabinoside (Tokyo Chemical Industry) was added to the medium to remove mitotic non‐muscle cells. For MG132 treatment, MG132 (Peptide Institute) was added to the differentiation medium at a final concentration of 1 µM for 16 h.

### 2.3 cDNA constructs

Mouse cDNAs for *Myh1* (85‐5913 in NM_030679), *Myh3* (45–5868 in NM_001099635), and *Myh7* (142‐5949 in NM_080728) were cloned into the peGFP‐C1 vector (TAKARA BIO). Mouse cDNA for *Neurl2* (Ozz, 157‐1014 in NM_001082974) was cloned into the pmCherry vector (TAKARA BIO). The inserted sequences of all constructs were verified with a 3730 DNA Analyzer (Applied Biosystems).

### 2.4 Real‐time quantitative PCR assay (RT‐qPCR)

Total RNA was isolated from differentiated muscle cells using ISOGEN (Nippon Gene) according to the manufacturer's instructions. The first strand cDNA was synthesized using ReverTra Ace (Toyobo). RT‐qPCR was performed with a CFX96 Real‐Time PCR Detection System (Bio‐Rad) using the QuantiTect SYBR Green PCR System (Qiagen). The primer pairs used in this study are listed in Table 1. PCR conditions were as follows: 15 min at 95°C, 45 cycles of 15 s at 94°C, 30 sec at 65°C and 30 s at 72°C. GAPDH was used as an internal control.

**TABLE 1:** Primer list for RT‐qPCR

Name	Sequence
cGapdh	fw: 5′‐CAACTTTGGCATTGTGGAGGGTCTTATGAC‐3′ rv: 5′‐AAACAAGCTTGACGAAATGGTCATTCAGTG‐3′
cOzz (Neurl2)	fw: 5′‐CTCCTGGATGAGCTGTACCGCAC‐3′ rv: 5′‐GAAGTGACCCTTGAAGGCCATC‐3′
mOzz (Neurl2)	fw: 5′‐TCTAGTGGAAATTGAGGAAAAAGAGCTGGG‐3′ rv: 5′‐CATGTCCTCCCCGTTGATGATGATGTG‐3′
GFP	fw: 5′‐CTACGGCAAGCTGACCCTGAAGTTCATC‐3′ rv: 5′‐CTCGATGTTGTGGCGGATCTTGAAGTTCAC‐3′
cMurf1 (Trim63)	fw: 5′‐CTCTGTGCACGTTTTGATGCGTTCTCA‐3′ rv: 5′‐ATAGAAAAGTGTCCTGTACTGGAGCTGGAT‐3′
cMurf2 (Trim55)	fw: 5′‐CAGCAATGACAGAGTACAAGGGATAGTCAC‐3′ rv: 5′‐GCCTCCTCCTCTCTGTCAAAATCAATTTCT‐3′
cMurf3 (Trim54)	fw: 5′‐GAAGAAGATCACAGACATGTCCAAGGTGTC‐3′ rv: 5′‐CTTCATAGCAGTCATAGGGCTGTAGGGTTT‐3′

c and m indicate chicken and mouse, respectively.

### 2.5 Fluorescence recovery after photobleaching (FRAP) assay

The FRAP assay was performed using transfected myotubes cultured on glass‐based dishes (IWAKI, Shizuoka, Japan) with a Leica TCS SP5 (Leica microsystem) as described previously (Ojima et al., 2015). Cells were incubated at 37°C and 5% CO_2_ on the microscope incubation system (TokaiHit) during FRAP experiments. The emission wavelengths of eGFP and mCherry were 488 and 543 nm, respectively, and band‐pass filters were 500–540 nm and 600–700 nm, respectively. A 100 µm^2^ region of interest (ROI) was selected in each cell. The fluorescence of ROIs was bleached by exposure to an argon laser at a strength of 100% for 30 s. Fluorescence recovery was monitored every hour after photobleaching. The fluorescence intensity of the ROI was quantified and normalized to that of the non‐bleaching area at each time point. The normalized fluorescence intensities were used in the following exponential curve fitting formula calculated by ImageJ 1.52a (National Institutes of Health) software:

FI = Mf * (1 − e^(−b*t)^) + c, where FI is the normalized fluorescence intensity, Mf is the mobile fraction, b is the speed constant, c is the fluorescence intensity after bleaching, and t is the elapsed time after beaching.

Mf was used for the maximum value of fluorescence intensity change. The 5% recovery time (5% Rt) indicated the time required to reach a fluorescence recovery of 5% of pre‐bleaching intensity, and was calculated from In[Mf/(Mf‐0.05)]/b. The 5% Rt was used for the parameter of speed for fluorescence recovery at the initial rise time.

### 2.6 Immunoprecipitation and western blotting

Immunoprecipitation samples were obtained from cells co‐transfected with eGFP‐Myh3 and mCherry‐Ozz. To increase the amount of ubiquitinated proteins, myotubes were treated with 1 µM MG132, a proteasome inhibitor, for 16 h. Immunoprecipitation was conducted under denature condition (Koyama et al., 2008). The cytosolic fraction was obtained by treating cells with modified chemical skinned‐fiber buffer [10 mM Tris‐HCl pH 7.5, 150 mM CsCl, 1 mM EDTA Cs, 0.5% (v/v) Triton X‐100, 0.1% (w/v) sodium dodecyl sulfate, 2% protease inhibitor cocktail (Sigma‐Aldrich), 0.06 mM leupeptin (Peptide Institute), 0.7 µM calpastatin (Takara Bio), and 25 µM MG132 (Peptide Institute)] for 30 min at 4℃. To remove cellular debris, the soluble fraction was centrifuged at 1500 g for 5 min. The supernatant was further centrifuged at 20,000 *g* for 20 min to obtain the cytosolic fraction. Precipitation fraction was prepared from myotubes after chemical skinned‐fiber buffer treatment. Skinned myotubes were collected in high‐salt lysis buffer [10 mM Tris‐HCl pH 7.6, 0.6 M CsCl, 1 mM EDTA Cs, 0.5% (v/v) Triton X‐100, 0.1% (w/v) sodium dodecyl sulfate, 2% protease inhibitor cocktail (Sigma‐Aldrich), 0.06 mM Leupeptin, 0.7 µM Calpastatin, 25 µM MG132]. Samples were homogenized with 27‐G syringes and rotated at 4℃ for 15 min to solubilize myosin. After centrifugation at 20,000 *g* for 20 min, the high‐salt soluble fraction was used as a myofibril fraction. The cytosolic and the myofibril samples were pre‐treated with agarose‐conjugated protein G (Santa Cruz Biotechnology) for 1 h to remove nonspecific proteins bound to the agarose beads. The supernatants were then reacted with anti‐myosin antibody (1:100; clone MF20, R&D Systems, Minneapolis, MN, USA) followed by protein‐G‐conjugated agarose beads at 4℃ for 1 h. The beads were washed with modified chemical skinned fiber buffer five times and then boiled with Laemmli sample buffer [125 mM Tris‐HCl pH 6.8, 20% (v/v) glycerol, 4% (w/v) SDS, 0.02% (w/v) bromophenol blue, and 0.2 M dithiothreitol] at 95℃ for 5 min.

### 2.7 Western blotting

Samples were analyzed by sodium dodecyl sulfate–polyacrylamide gel electrophoresis (SDS‐PAGE) followed by transfer to PVDF membranes (Merck). Membranes were blocked with 0.5% (w/v) bovine serum albumin (FUJIFILM Wako Pure Chemical Corporation, Osaka, Japan) at room temperature for 30 min and then incubated at 4℃ for 16 h with one of the following antibodies: mouse anti‐myosin heavy chain (1:1000; clone MF20, R&D Systems), mouse anti‐ubiquitin (1:1,000; clone VB2880, VIVA Bioscience), and mouse anti‐mRFP (1:1,000; clone mix of 1G9 and 3B5, MBL). Following incubation with peroxidase‐conjugated mouse secondary antibodies (NICHIREI BIOSCIENCES), bands were visualized with the POD immunostain kit (FUJIFILM Wako Pure Chemical Corporation) or ECL Western Blotting Detection Reagents (GE HealthCare). For band quantification, signal intensities of bands were analyzed by ImageJ 1.52a (National Institutes of Health). As T. Nastasi, et al (Nastasi et al., 2004) described previously, the ratios of Ub‐Myh band intensity to Myh band intensity were calculated as follows: (the signal intensity of Ub‐Myh band/ the lower Ig band) / (the signal intensity of Myh band / the lower Ig band). The ratios were then normalized with values of controls.

### 2.8 Immunofluorescence staining

Myotubes cultured in 8‐well chambers (Thermo Fisher Scientific) were treated with MG132 for 16 h before fixing and washing with 0.5% (v/v) Triton X‐100 in PBS. Then, myotubes were blocked with 0.5% (w/v) bovine serum albumin (FUJIFILM Wako Pure Chemical Corporation) at room temperature for 30 min. After blocking, myotubes were reacted with mouse anti‐ubiquitin antibody (1:100; clone VU‐1, LifeSensors, Malvern, PA, USA) and rabbit anti‐pan sarcomeric myosin heavy chain antibody (1:4,000; kindly gifted by Prof. Howard Holtzer, Univ. Penn), followed by incubation with FITC and TRITC secondary antibodies (anti‐Rabbit, 611‐102‐122, Rockland, PA, USA) (anti‐mouse, #55527, Cappel, NC, USA). Specimens were mounted with mounting media (Thermo Fisher Scientific) and observed using the Leica TCS SP5 (Leica microsystem).

### 2.9 Statistics

All data are expressed as the mean ± standard error (SE) with individual datapoints. Control and each trial data were compared using the t‐test. Statistical significance was set at *p* < 0.05. All statistical tests were performed using EZR on R commander ver. 1.40.

## 3 RESULTS

### 3.1 Overexpressed Ozz localizes to the cytosol and does not alter the expression levels of eGFP‐Myh3 and endogenous Ozz

**FIGURE 1:**
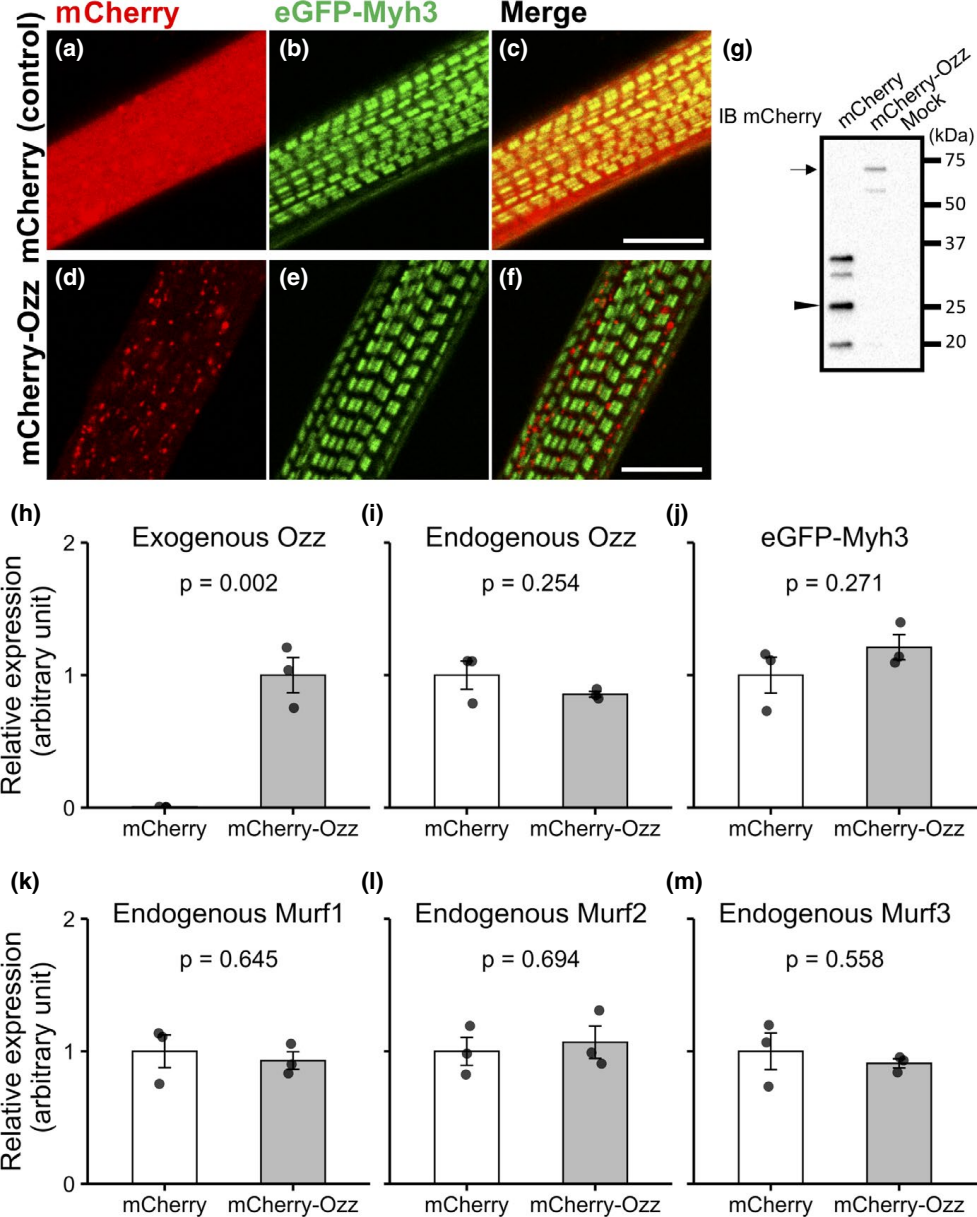
Localization and expression level of exogenously expressed mCherry‐Ozz in muscle cells. (a–f) Localization of exogenously expressed eGFP‐Myh3 and mCherry‐Ozz observed by confocal microscopy. eGFP‐Myh3 localized to the A‐bands of myotubes (green). mCherry‐Ozz was distributed as small puncta in myotubes (red in d and f). mCherry expressed alone as a control showed a diffuse distribution in myotubes (red in a and c). c and f are merged images of a and b, and d and e, respectively. Bars, 10 µm. (g) The expression of mCherry‐Ozz and mCherry proteins in myotubes was detected by western blotting. An arrow and an arrowhead indicate the bands corresponding to mCherry‐Ozz and mCherry, respectively. (h–m) The expression levels of mCherry‐Ozz, endogenous Ozz, eGFP‐Myh3, and endogenous Murf1~3 were quantified by RT‐qPCR in myotubes co‐expressing eGFP‐Myh3 and either mCherry (control) or mCherry‐Ozz. GAPDH was used as the internal control, and the relative expression levels were calculated as a ratio of the signal intensity of the target gene to the signal intensity of GAPDH. Fold change was normalized to the mCherry‐Ozz group (h) or mCherry group (i‐m). Values represent the mean ± SE. n = 3 for each group

To explore the involvement of the UPS in myosin replacement, we examined the role of the Myh3‐specific E3 ligase Ozz (Campos et al., 2010). First, we tested whether exogenously expressed Ozz localized to specific regions in myotubes. Cultured skeletal muscle cells were transfected with constructs encoding eGFP‐Myh3 and mCherry‐Ozz. Although exogenously expressed mCherry‐Ozz was mainly distributed as puncta between myofibrils, the mCherry‐Ozz puncta did not affect the structure of eGFP‐positive thick filaments (Figure 1a–f). Figure 1G shows that the molecular weight of exogenously expressed mCherry‐Ozz in myotubes. The effect of exogenously overexpressed Ozz on the expression levels of endogenous Ozz and exogenous eGFP‐Myh3 was examined by RT‐qPCR. The results showed that the mRNA expression levels of endogenous Ozz did not differ significantly between the Ozz overexpressing and the control groups (Figure 1h–i), and eGFP‐Myh3 was expressed at comparable levels in the two groups (Figure 1j). Furthermore, we confirmed that Ozz overexpression did not change the expression levels of endogenous Murf family members (Figure 1k–m). These results indicate that exogenously expressed mCherry‐Ozz did not alter the expression levels of endogenous Ozz and Murf family, and exogenous eGFP‐Myh.

### 3.2 mCherry‐Ozz overexpression decreases the replacement rate of the eGFP‐Myh3

**FIGURE 2:**
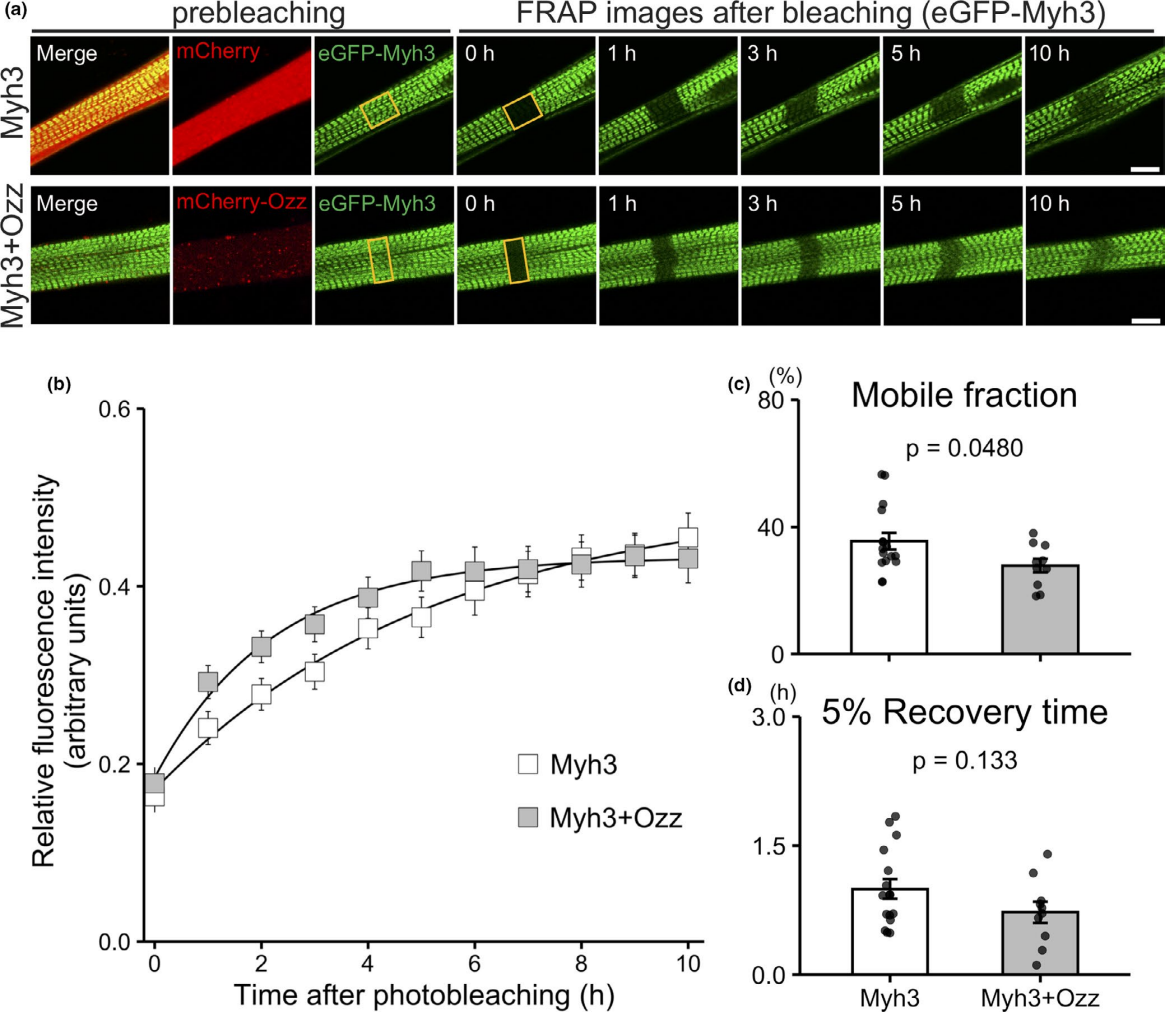
Fluorescence recovery of eGFP‐Myh3 in myotubes overexpressing mCherry‐Ozz. (a) Representative FRAP images of myotubes co‐expressing eGFP‐Myh3 (green) and either mCherry or mCherry‐Ozz (red). Images were obtained at 1‐h intervals after photobleaching. Bleached areas are indicated by yellow rectangles. (b) Normalized fluorescence intensities of eGFP‐Myh3 were obtained from myotubes co‐expressing eGFP‐Myh3 and mCherry (control) or mCherry‐Ozz. (c and d) Mobile fractions and 5% fluorescence recovery times were calculated from the graph as described in the Materials and Methods section. Values represent the mean ± SE. Myh3, n = 16. Myh3+Ozz, n = 10

To examine effect of Ozz overexpression on eGFP‐Myh3 replacement, myotubes co‐expressing eGFP‐Myh3 and mCherry‐Ozz were analyzed using the FRAP assay. Changes in fluorescence intensity at the bleaching area were measured at 1‐h intervals after fluorescence bleaching to determine the fluorescence recovery of eGFP‐Myh3 in myotubes overexpressing mCherry or mCherry‐Ozz (Figure 2a). The eGFP‐Myh3 fluorescent recovery rate was lower in myotubes expressing mCherry‐Ozz than in control myotubes (Mf 27.8% ±2.1% in Myh3+Ozz vs. 35.6% ±2.6% in Myh3, *p* = 0.048), although the recovery time of eGFP‐Myh3 did not differ significantly from that of the control (5% Rt 0.73 ± 0.12 h in Myh3+Ozz vs. 0.99 ± 0.11 h in Myh3, *p* = 0.133) (Figure 2b–d). These results indicate that overexpression of Ozz decreased the eGFP‐Myh3 replacement rate in myofibrils.

### 3.3 Ozz does not alter the replacement rate of eGFP‐Myh1 and eGFP‐Myh7

**FIGURE 3:**
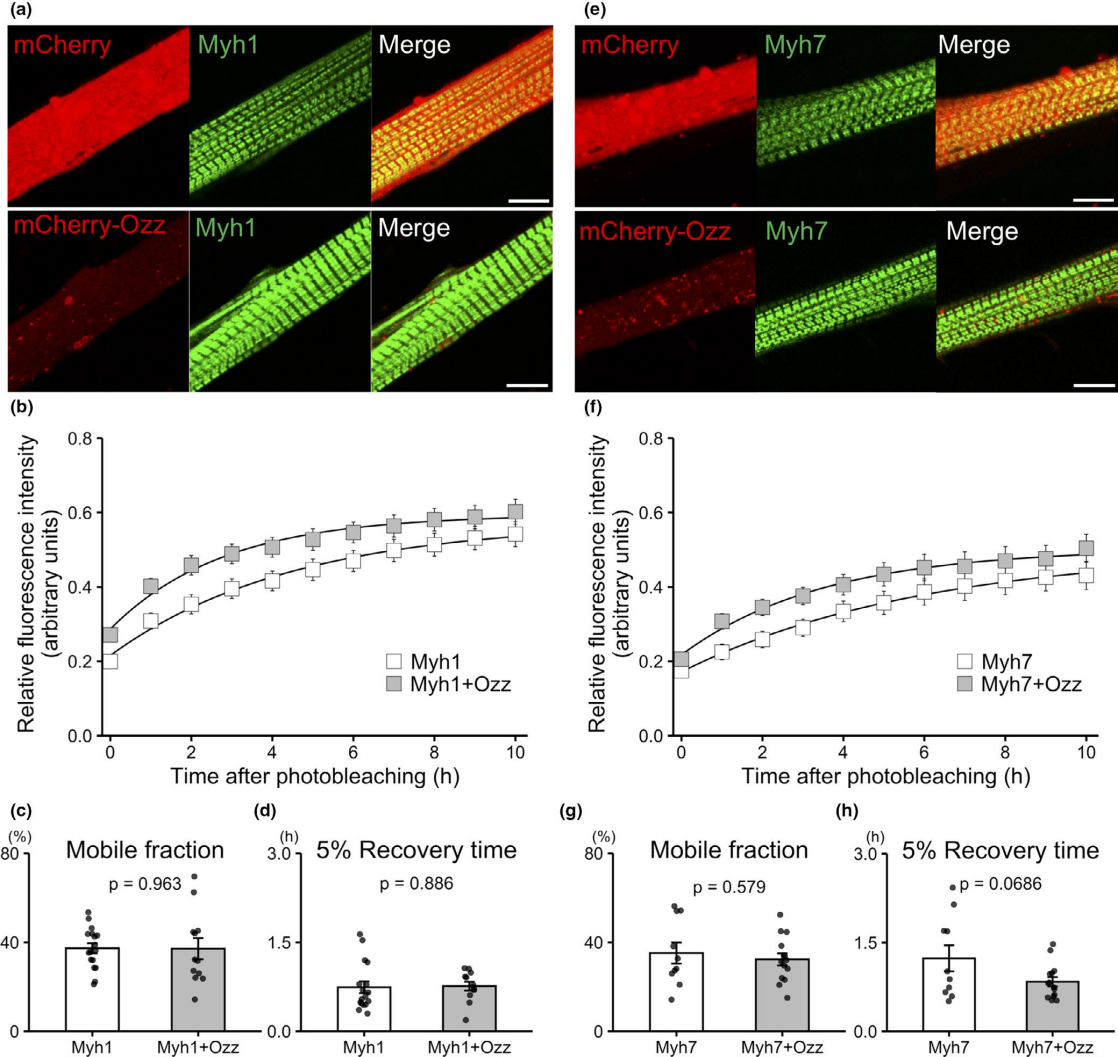
Fluorescence recovery of eGFP‐Myh1 and eGFP‐Myh7 in myotubes overexpressing mCherry‐Ozz. (a, e) Localization of exogenously co‐expressed eGFP‐Myh1 and either mCherry or mCherry‐Ozz in A, and eGFP‐Myh7 and either mCherry‐Ozz or mCherry‐Ozz in E. eGFP‐Myh1 and eGFP‐Myh7 were localized to the A‐bands of myotubes (green). mCherry and mCherry‐Ozz were distributed diffusely or as small puncta in myotubes (red). Bars, 10 µm. (b and f) Fluorescence recovery of eGFP‐Myh1 in b or eGFP‐Myh7 in f was measured in myotubes co‐expressing mCherry‐Ozz (grey squares) or mCherry (white squares). (c and g) Mobile fractions were calculated from the fluorescence recovery curves of eGFP‐Myh1 or eGFP‐Myh7. (d and h) The 5% recovery times were calculated from the fluorescence recovery curves of eGFP‐Myh1 or eGFP‐Myh7. Data are expressed as the mean ± SE. Myh1, n = 17. Myh1+Ozz, n = 12. Myh7, n = 10. Myh7+Ozz, n = 14

Because overexpression of Ozz decreased the replacement rate of eGFP‐Myh3 (the embryonic myosin isoform), we tested the effects of Ozz on the replacement rates of other myosin isoforms such as Myh1 and Myh7. eGFP‐Myh1 and eGFP‐Myh7 were localized to the A‐bands in myotubes overexpressing mCherry and mCherry‐Ozz (Figure 3a,e). The results of the FRAP assays showed that the replacement rate of eGFP‐Myh1 in myotubes co‐expressing mCherry‐Ozz did not differ significantly from that in control myotubes overexpressing mCherry (Figure 3a–d). Similarly, Ozz overexpression did not decrease the replacement rate of eGFP‐Myh7 in myotubes overexpressing mCherry‐Ozz (Figure 3e–h). These results indicate that overexpression of Ozz specifically reduced the replacement rate of eGFP‐Myh3, whereas it had no effect on eGFP‐Myh1 and eGFP‐Myh7.

### 3.4 Ozz induces myosin ubiquitination in myotubes

**FIGURE 4:**
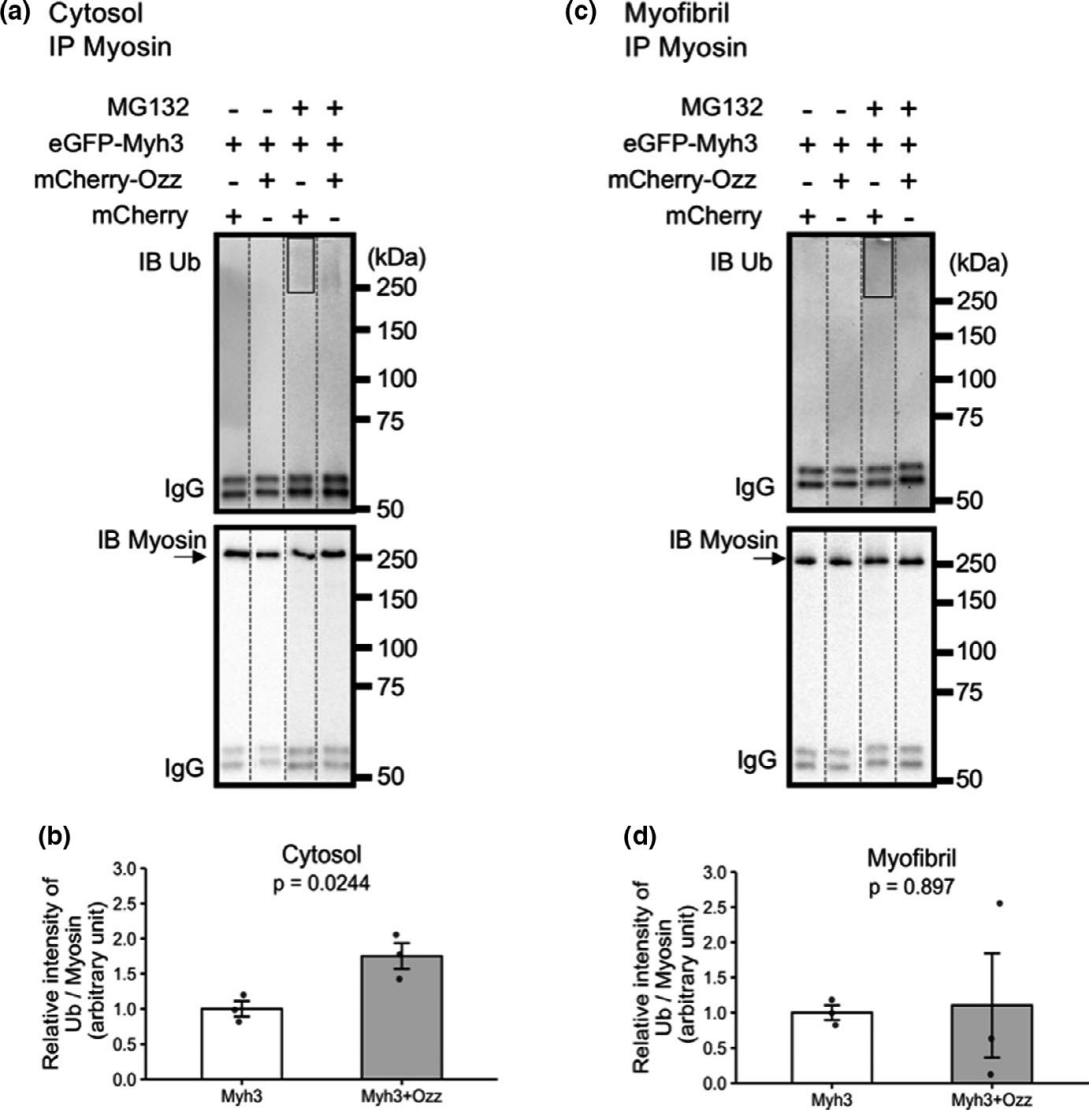
Enhanced ubiquitination of Myh in muscle cells overexpressing mCherry‐Ozz. (a and c) eGFP‐Myh3 and mCherry or mCherry‐Ozz were co‐expressed in muscle cells treated with or without MG132. Cytosolic in A and myofibril in C fractions were obtained as low and high‐salt buffer soluble fractions. Samples were immunoprecipitated with anti‐Myh antibody (MF20), and subjected to western blotting against anti‐Myh or anti‐ubiquitin antibody. Arrows indicate bands corresponding to Myh. (b and d) The band intensity of ubiquitinated Myh in MG132‐treated samples was quantified in the area indicated by rectangles in A and C. Relative intensity of Ub‐Myh to Myh was calculated as described in the Materials and methods section. Data are expressed as the mean ± SE. n = 3 for each group

Since Ozz functions as a Myh3‐specific Ub ligase (Campos et al., 2010), we hypothesized that the Ozz‐induced decrease in the myosin replacement rate was caused by the effect of Ozz overexpression on targeting eGFP‐Myh3 for degradation. To address this, cultured muscle cells were separated into a cytosolic fraction and a myofibril fraction, and ubiquitinated myosin was detected by immunoprecipitation and immunoblotting in each fraction. Cells were treated with MG132 to detect ubiquitinated myosin, because ubiquitin bands scarcely detected without MG132 treatment (Figure 4a,c). In the cytosolic fraction, the relative signal intensity of ubiquitinated‐Myh detected by immunoblotting was significantly higher in muscle cells overexpressing Ozz than in control muscle cells (1.00 ± 0.11 in Myh3, 1.75 ± 0.18 in Myh3+Ozz) (Figure 4a,b). In the myofibril fraction, the signal intensity of ubiquitinated‐Myh had no difference (1.00 ± 0.10 in Myh3, 1.10 ± 0.74 in Myh3+Ozz) (Figure 4c,d). Intriguingly, the band intensities of anti‐ubiquitin antibody were scarcely detectable in the myofibril fraction with MG132. These results indicate that overexpression of Ozz increased Myh ubiquitination and probably enhanced UPS‐mediated degradation in the cytosol.

### 3.5 Ubiquitinated‐Myh is abundant in the cytosolic fraction and is not replaced in the thick filament

**FIGURE 5:**
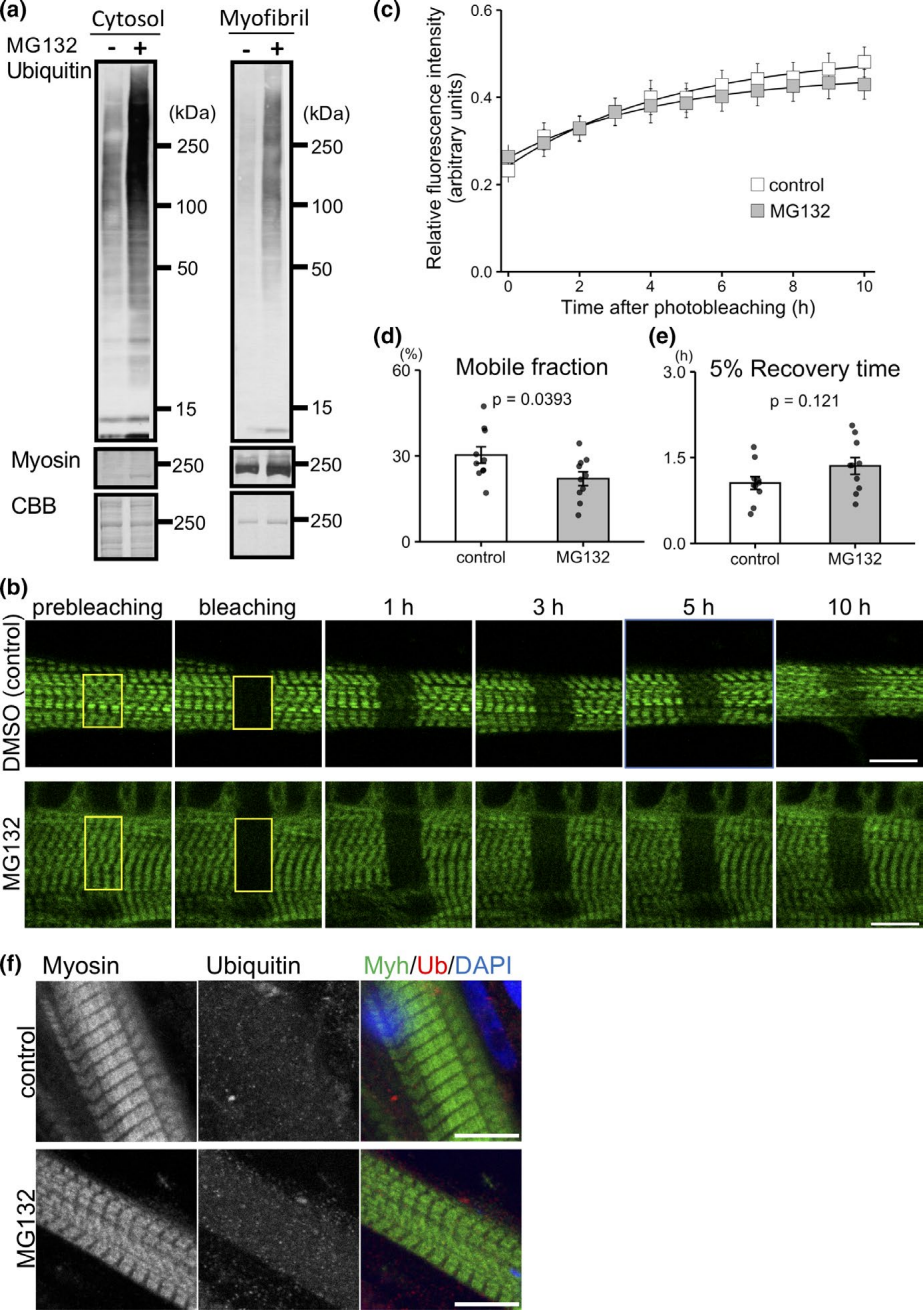
Fluorescence recovery of eGFP‐Myh3 in myotubes treated with MG132. (a) Ubiquitinated proteins were detected by western blotting. Samples were obtained from muscle cells treated with or without MG132. (b) Representative FRAP images of in myotubes expressing eGFP‐Myh3 in the presence of DMSO (control) or MG132. Images were obtained at 1‐h intervals after photobleaching. Bleached areas are indicated by yellow rectangles. (c) Normalized fluorescence intensities of eGFP‐Myh3 were obtained from DMSO (control) or MG132‐treated myotubes. (d and e) Mobile fractions and 5% fluorescence recovery times were calculated. Values represent the mean ± SE. n = 10 for each group. (f) Myotubes treated with or without MG132 were stained with anti‐myosin antibody (green) and anti‐ubiquitin antibody (red). Nuclei were visualized with DAPI. Bars, 10 µm

The present results indicate that Ozz decreased the Myh3 replacement rate by promoting myosin degradation. We, therefore, explored the effect of inhibition of protein degradation on myosin replacement. To address this, cultured muscle cells were treated with the proteasome inhibitor MG132, which does not inhibit ubiquitination but does inhibit protein degradation by UPS. MG132 treatment increased the level of ubiquitinated proteins in the cytosolic and myofibril fractions (Figure 5a). To test the effect of inhibition of protein degradation on myosin replacement, myotubes expressing eGFP‐Myh3 in the presence or absence of MG132 were analyzed using the FRAP assay. eGFP‐Myh3 was detected in the thick filaments of myofibrils in myotubes treated with MG132, indicating that MG132 did not disrupt the myofibrils structure (Figure 5b). FRAP assays indicated that the replacement rate of eGFP‐Myh3 was significantly lower in MG132‐treated myotubes than in the control group (Figure 5c–e). These results suggest that ubiquitinated myosin is not frequently replaced in myofibrils in the presence of MG132. Next, we examined the localization of ubiquitin in myotubes treated with or without MG132. Ubiquitin was not detected in the thick filaments, but rather in the cytoplasm in a diffuse pattern or in small puncta. This microscopic observation was consistent with the immunoblot data and indicated that ubiquitinated myosin predominantly localized to the cytoplasm, suggesting that ubiquitinated myosin is not the primary source for myosin substitution in myofibrils (Figure 5f).

## 4 DISCUSSION

In this study, overexpression of Ozz decreased the Myh3 replacement rate in myotubes probably through promoting the ubiquitination and degradation of Myh3. MG132 treatment led to the accumulation of ubiquitinated‐Myh in the cytosol rather than in the myofibrils of myotubes, suggesting that the myosin in myofibrils was not replaced by ubiquitinated myosin. Myosin in the cytosol is one of the main source for myosin replacement (Ojima et al., 2017). The decrease of replaceable myosin in the cytoplasm of myotubes caused by increased ubiquitination by Ozz and UPS‐mediated degradation led to a low myosin replacement rate in myofibrils.

In the present study, Ozz overexpression specifically decreased eGFP‐Myh3 replacement rate. Because Myh3 is a substrate of the E3 ligase Ozz (Campos et al., 2010), we hypothesized that Ozz overexpression promoted the ubiquitination and proteasomal degradation of eGFP‐Myh3. The increased degradation rate of Myh3 may have limited the supply of Myh3 for myosin substitution, thereby decreasing the eGFP‐Myh3 replacement rate. This hypothesis was supported by the results showing that Ozz overexpression increased myosin ubiquitination in the cytosol, and not in the myofibrils (Figure 4). The finding that overexpressed mCherry‐Ozz did not localize to the myofibrils suggests that Ozz promotes the degradation of myosin in the soluble cytosolic fraction rather than in the myofibril. This is similar to other E3s such as Murfs (Cohen et al., 2009; Solomon & Goldberg, 1996), which capture myosin in the soluble cytosolic fraction of skeletal muscle cells. In addition, Ozz may target impaired or expendable myosin for degradation in the cytosolic fraction rather than in the myofibrils. It is unlikely that Ozz captures impaired or expendable myosins in the thick filaments because Ozz interacts with myosin through the assembly competence domain of Myh3 (1873–1901 aar) (Campos et al., 2010), which is essential for the formation of the thick filaments and is concealed in the thick filament (Sohn et al., 1997). Therefore, interaction with Ozz may interfere with the incorporation of the Myh–Ozz complex into the thick filaments, leading to a decrease in the myosin replacement rate in myotubes overexpressing Ozz.

Another possible explanation for the reduction in Myh3 replacement rate in myotubes overexpressing Ozz and myotubes treated with MG132 is that ubiquitination of Myh3 changes its biochemical properties. Ubiquitination is a post‐translational modification (Passmore & Barford, 2004) that functions not only as a tag for degradation, but also as a modification factor for protein solubility (Dao et al., 2018; Sharkey et al., 2018). Biochemical studies demonstrated that in the presence of MG132, ubiquitinated myosin accumulated in the cytosol rather than in myofibrils. Furthermore, immunofluorescence studies showed that ubiquitin did not co‐localize with myosin in the thick filaments but was instead distributed diffusely in the cytosol (Figure 5f). The modification of myosin with ubiquitin may increase its solubility to prevent the formation of aggregates, which form under physiological ionic conditions (Davis, 1988). Although MG132‐treated myotubes contained ubiquitinated myosin in the cytosol, the myosin replacement rate was slow. Thus, it is likely that ubiquitinated myosin in the cytosol is not replaceable in the thick filaments. Moreover, ubiquitinated myosin scarcely accumulated in the absence of MG132 in both the cytosolic and myofibril fraction, suggesting that ubiquitinated myosin is immediately disassembled (Figure 4a,c). The increase in myosin solubility caused by ubiquitination may improve the degradation efficiency by suppressing myosin insertion into myofibrils. Taken together, these observations indicate that ubiquitinated myosin tends to stay in the cytosol rather than being incorporated into the thick filaments.

The myosin replacement rates differed between slow and fast myosins, i.e., eGFP‐Myh7 showed the slowest myosin replacement speed (5% Rt 1.23 h ± 0.22 h) among three myosin isoforms (0.74 ± 0.10 h in eGFP‐Myh1, and 1.00 h ± 0.11 h in eGFP‐Myh3) and replacement speed is statistically different between Myh1 and Myh7 (Figures 2 and 3). Because the intracellular environment was identical in myotubes expressing eGFP‐Myh7, eGFP‐Myh1, or eGFP‐Myh3, these results suggest that differences in the replacement rate were caused by the intrinsic properties of the different myosin isoforms. The amino acid sequence homology of the myosin isoforms is relatively high: Myh7 and Myh1, 81.9%. However, amino acid sequence variations are found in the tail region of Myhs, which is pivotal role in myosin filament assembly. Because amino acid composition determines the myosin axial arrangement (Atkinson & Stewart, 1992; Sohn et al., 1997), differences in the composition of the tail among myosin isoforms may change the assembly properties of myosin.

As the UPS was proposed to regulate the myosin replacement rate, it is possible that the different isoforms of myosin are differentially regulated by the UPS. The Murf family (Murf1, Murf2, and Murf3) of muscle‐specific ubiquitin ligases is myosin‐associated protein (Cohen et al., 2009; Fielitz et al., 2007; Pizon et al., 2002). Murfs work cooperatively, and double knockout of Murf1 and Murf3 or Murf2 and Murf3 in mice causes myosin aggregation and myopathy (Fielitz et al., 2007; Lodka et al., 2016). However, Murf expression is muscle type‐specific in vivo: Murf1 is predominantly expressed in slow type myofibers, Murf2 is expressed in fast type myofibers, and Murf3 is expressed ubiquitously (Moriscot et al., 2010; Perera et al., 2012). Murfs may preferentially recognize specific myosin isoforms, which may affect the myosin replacement rate, similar to the effect of Ozz on decreasing the Myh3 replacement rate.

**FIGURE 6:**
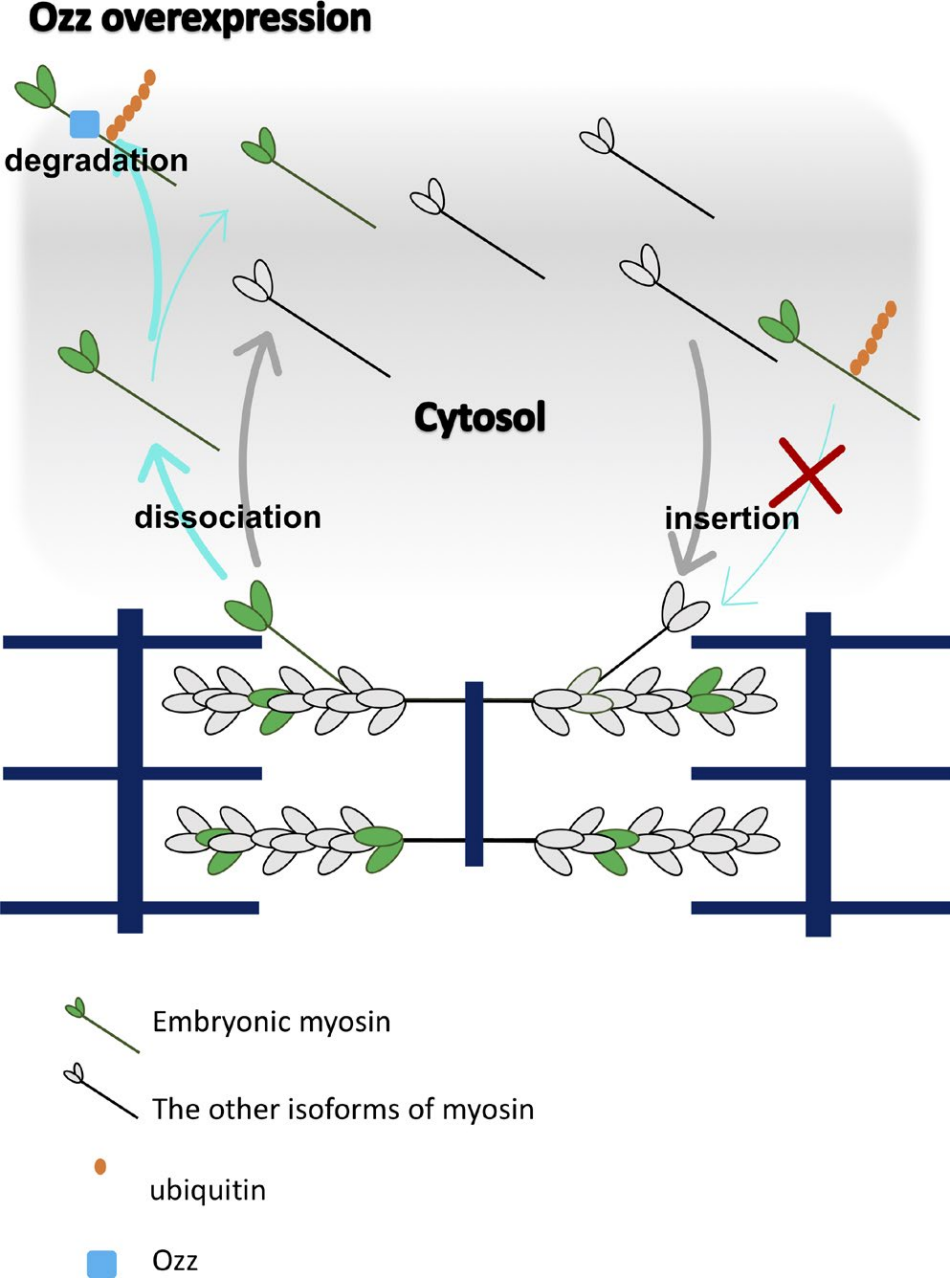
A model of myosin replacement in the presence of overexpressed Ozz. Ozz overexpression in myotubes promotes ubiquitination and degradation of embryonic myosin (Myh3) in the cytosol, and/or addition of ubiquitin chain to Myh3 decrease the insertion of Myh3 into the thick filaments. Replacement rates of the other myosin isoforms are not affected by Ozz overexpression. Blue and grey arrows indicate flows of embryonic and the other myosin isoforms, respectively

We propose a model of myosin replacement in the presence of Ozz overexpression (Figure 6). Overexpression of Ozz increases Myh3 ubiquitination and probably degradation, which decreases the eGFP‐Myh3 replacement rate. In the differentiation stage and during muscle remodeling, Myh in the thick filaments shifts from the embryonic to the adult isoform (Schiaffino et al., 2015). The up‐regulation of Ozz during muscle differentiation (Nastasi et al., 2004) results in the selective degradation of ubiquitinated Myh3, which promotes an isoform shift from embryonic myosin to neonatal/adult myosins (Campos et al., 2010). Therefore, Ozz may regulate the myosin replacement rate by catalyzing the modification of Myh3 to ensure an efficient myosin isoform shift in the thick filaments.

## CONFLICT OF INTEREST

The authors have no conflicts of interest to declare.

## AUTHOR CONTRIBUTIONS

E.I., K.O., T.S., K.K., and T.N. designed the research; E.I. and K.O. performed the experiments; E.I., K.O., and S.M. analyzed data; E.I., K.O., and T.N. wrote the manuscript.
